# Patient-Reported Outcome Measures of Utilizing Person-Generated Health Data in the Case of Simulated Stroke Rehabilitation: Development Method

**DOI:** 10.2196/16827

**Published:** 2020-05-07

**Authors:** Gerardo Luis Dimaguila, Kathleen Gray, Mark Merolli

**Affiliations:** 1 School of Computing and Information Systems University of Melbourne Melbourne Australia; 2 Centre for Digital Transformation of Health University of Melbourne Melbourne Australia

**Keywords:** patient monitoring, patient reported outcome measures, patient generated health data, person generated health data, questionnaire design, telemedicine

## Abstract

**Background:**

Person-generated health data (PGHD) are health data that people generate, record, and analyze for themselves. Although the health benefits of PGHD use have been reported, there is no systematic way for patients to measure and report the health effects they experience from using their PGHD. Patient-reported outcome measures (PROMs) allow patients to systematically self-report their outcomes of a health care service. They generate first-hand evidence of the impact of health care services and are able to reflect the real-world diversity of actual patients and management approaches. Therefore, this paper argues that a PROM of utilizing PGHD, or PROM-PGHD, is necessary to help build evidence-based practice in clinical work with PGHD.

**Objective:**

This paper aims to describe a method for developing PROMs for people who are using PGHD in conjunction with their clinical care—*PROM-PGHD*, and the method is illustrated through a case study.

**Methods:**

The five-step qualitative item review (QIR) method was augmented to guide the development of a PROM-PGHD. However, using QIR as a guide to develop a PROM-PGHD requires additional socio-technical consideration of the PGHD and the health technologies from which they are produced. Therefore, the QIR method is augmented for developing a PROM-PGHD, resulting in the PROM-PGHD development method.

**Results:**

A worked example was used to illustrate how the PROM-PGHD development method may be used systematically to develop PROMs applicable across a range of PGHD technology types used in relation to various health conditions.

**Conclusions:**

This paper describes and illustrates a method for developing a PROM-PGHD, which may be applied to many different cases of health conditions and technology categories. When applied to other cases of health conditions and technology categories, the method could have broad relevance for evidence-based practice in clinical work with PGHD.

## Introduction

### Understanding the Effects of Person-Generated Health Data

Person- or patient-generated health data (PGHD) are health, wellness, and other biometric data that people generate, record, and analyze for themselves [1]. Examples of technologies that support PGHD include Web-based journaling tools, activity-tracking devices or mobile apps, networked health data–gathering devices such as weighing scales, and simulated rehabilitation technologies. Patients who use PGHD-enabled technologies may experience positive, negative, or nil effects. PGHD use has been reported to increase patients’ interest in their own health care processes [2-4] and the management of their own health status [5]. It is known that when patients understand their illness, they may become active problem solvers and improve their health behavior [6]. However, PGHD use can also cause feelings of frustration and discouragement [7], and may even make some patients feel excluded from the benefits of PGHD use [5].

Although such varying health effects of PGHD use have been reported for a variety of health conditions and technology types, there is no systematic way for patients to measure and report health effects that they experience from utilizing their PGHD—whether positive, negative, or nil. This may hamper the integration of PGHD into clinical workflows [1]. In addition, PGHD technologies may be designed to support clinicians’ utilization of these data at the expense of functionality that supports patients to use their data for self-management and shared decision making [8]. Thus, it is necessary to consider the patient’s perspective in the design and development of health technologies [9], particularly those that generate PGHD [8].

### Patient-Reported Outcome Measures

In health care services and interventions in general, the measurement of effects on patients, by patients themselves, is not new. Patient-reported outcomes are self-reported status updates of a patient’s health condition, experience with an illness, or treatment without additional interpretation of the report, for example, by clinicians [10-12]. They may be used to indicate health status, such as state of a disease, at a single point in time, and any changes over time from previous patient-reported outcomes [10,13].

Standardized instruments that measure patient-reported outcomes, Patient-Reported Outcome Measures (PROMs) contribute to a more precise evaluation of the effects of a variety of health interventions and improve the evidence base in many areas of clinical care [14,15]. PROMs are used to determine the effectiveness of health care practices and to set standards for health care providers’ performance, and their importance is highlighted by several national projects [15,16].

PROMs are developed systematically [10,11,13], and this formalism makes PROMs valuable to complement clinician-reported outcome measures used in reporting as part of standardized treatment assessments, such as clinician assessments of patient health, health outcome indicators collected routinely by health care organizations, and physiological or other biomedical indicators [15]. Their utility in generating first-hand evidence of the impact of health care services enables them to reflect the real-world diversity of actual patients and management approaches [17,18]. Thus, PROMs may provide a more comprehensive and accurate assessment of patient outcomes and the effectiveness of health care services and interventions [11,15,19,20].

### Patient-Reported Outcome Measures of Utilizing Person-Generated Health Data

A systematic way for patients to measure and self-report the health effects they experience from utilizing their PGHD is lacking. A PROM of utilizing PGHD, or PROM-PGHD, is necessary to help build evidence-based practice (EBP) in clinical work with PGHD. Measuring outcomes of PGHD utilization using PROMs has been suggested [21]. Patient participation is considered essential in developing PROMs [10,22,23], with nearly three-quarters of PROM-development papers including patients during the process [24]. Given PGHD’s person- or patient-centric approach to health data, it is useful and appropriate to involve patients in developing a standard way of using PROMs to capture the effects of PGHD. The participatory health paradigm recognizes the value of having patients contribute to the creation of knowledge in such ways [25].

PROMs-PGHD may deepen our understanding of how PGHD impact the health status and quality of life of patients, in an era of mobile and wearable remote patient monitoring [26]. PROMs-PGHD could also be used as a complement to existing clinician-reported and patient-reported outcomes, similar to how many PROMs are used alongside other health outcome indicators [15]. While many PROMs allow patients to report outcomes that correlate with their quantifiable PGHD [26], specific PROMs-PGHD would allow more direct self-reporting of the effects on patient health of utilizing PGHD. PROMs-PGHD could contribute to a more holistic and accurate assessment of whether and how patients’ use of PGHD from health self-monitoring technologies actually has health benefits. This would provide a triangulated measurement of patients’ experiences and outcomes resulting from their use of health information technology.

### Objective

The aim of this paper was to describe and illustrate a method for developing PROMs for people who are utilizing PGHD in conjunction with their clinical care—*PROM-PGHD*.

## Methods

This section reviews practices for developing PROMs, provides a rationale for the selection of the qualitative item review (QIR) method to develop a PROM-PGHD, and explains the need to augment QIR considering the socio-technical domains of health technologies.

### Patent-Reported Outcome Measure Development Practices

PROMs are developed in many different ways, but generally accepted elements in the process can be discerned [15]. Reviewing recognized methods for PROM development (Table 1) and their commonalities put into context the selection of a particular method to guide PROM-PGHD development.

**Table 1:** Patient-reported outcome measure (PROM) development: the best practice activities.

Number	Phases (review paper [23])	Steps (US Food and Drug Administration Guide [10])	Stages (Scientific Advisory Committee of the Medical Outcomes Trust [21,22,26])
1	Establish correct health outcomes to measure	Hypothesize conceptual framework Concepts hypothesized Target population and application of the PROM identified Literature or expert review conducted	Conceptual model for the PROM and its Initial Items are developed Includes literature review to identify existing PROMs within the target domain Interviews and/or focus groups with the target population, condition, or disease Identification of relevant areas as a basis for PROM development Pilot testing of initial PROM items on a small cohort of patients
2	Develop PROM items	Adjust conceptual framework and draft instrument Patient input obtained New PROM items generated Method of data collection/administration determined PROM draft items pilot tested	Revised PROM items from stage I are field-tested on a larger cohort of patients Results in further item revisions to improve item validity Reductions to eliminate redundancy, endorsement frequency, and absent data
3	Test the PROM items on comprehensibility and a range of psychometric criteria, for example, acceptability, internal consistency, and reliability	Confirm conceptual framework and assess other measurement properties Developed conceptual framework confirmed via a scoring rule PROM items assessed using psychometric criteria and finalized for content and format	Psychometric field-testing of the PROM being developed Resulting PROM administered to a large cohort of patients and tested based on a psychometric criterion, for example, acceptability, internal consistency, and reliability
4	N/A^a^	Collect, analyze, and interpret data Protocol and statistical plan for PROM data collection and analysis developed Product treatment responses evaluated and benefits interpreted	N/A
5	N/A	Modify instrument PROM items revised again using psychometric criteria PROM items translated and adapted culturally for multiple languages; this fifth step then leads back iteratively to the first step	N/A

^a^N/A: not applicable.

We found a scoping review of 189 PROM development papers from 1980 to 2014 that outlined the development processes of 193 PROMs retrieved from the PubMed, Cochrane Methodology, MEDLINE, and EMBASE databases [24]. This review noted that PROM development follows three broad, distinct phases, as shown in Table 1, although the review paper itself provided limited information on those phases. One of the included papers was the highly cited US Food and Drug Administration (FDA) industry guide to use PROMs for medical product labeling [10]. Many of the suggested activities in its first three steps align with the three phases described in the review paper [24]. However, the FDA guide suggests a more detailed, 5-step iterative process for developing PROMs [10], as shown in Table 1.

Another highly cited guide for PROM development, not included in the review paper, is that of the Scientific Advisory Committee (SAC) of the Medical Outcomes Trust [22]. This defines a set of attributes for developing and assessing instruments for measuring health status and quality of life, and recommends a 3-stage process for developing PROMs [23,27], as shown in Table 1.

We observed that the steps of the FDA guide [10] align with many of the activities outlined by the SAC [23,27], and consequently both align with the three phases described in the review paper (Table 2) [24]. This indicated consensus on the best practice in PROM development and gave us an understanding of what the developers of a PROM-PGHD must do so as to adhere to the best practice.

**Table 2:** Parallels between patient-reported outcome measure (PROM) development processes in the literature.

Phases (review paper [23]) and steps (US Food and Drug Administration Guide [10])		Stages (Scientific Advisory Committee of the Medical Outcomes Trust [21,26])
**Phase 1: Establish correct health outcomes to measure**		
	Step 1: Hypothesize conceptual framework	Stage I: Conceptual model for the PROM and its initial items are developed
**Phase 2: Develop PROM items**		
	Step 2: Adjust conceptual framework and draft instrument	Stage I: Conceptual model for the PROM and its initial items are developed
**Phase 3: Test the PROM items on comprehensibility and a range of psychometric criteria**		
	Step 3: Confirm conceptual framework and assess other measurement properties	Stage III: Psychometric field-testing of the PROM being developed
	Step 4: Collect, analyze, and interpret data	Stage III: Psychometric field-testing of the PROM being developed
	Step 5: Modify instrument	All stages: PROM item revision activities
	Iteration back to Step 1, with further testing	Stage II: Revised PROM items from Stage I are field-tested on a larger cohort of patients

### Qualitative Item Review

The systematic QIR process was designed to develop PROM items for the Patient Reported Outcomes Measurement Information System (PROMIS), a US National Institutes of Health initiative to provide a PROMs infrastructure for clinical research and practice [15,16]. QIR was intended to identify and develop items that could precisely estimate the traits being measured, and to represent the range of experiences relevant to the domains of interest. QIR is based on the best practices of PROM development and is committed to involving patients in the process, as described below. All of these factors make it suitable as a foundation for developing a PROM-PGHD.

PROM development falls within the participatory health paradigm, as the patient’s perspective is central to the value of PROMs [14]. Thus, patient participation should be deliberate in the development of a PROM-PGHD. QIR was developed with a commitment to involving patients, with a reference to the recommendation in the FDA guide [10]. It specifically suggests when and how patients are included in the development process. It also examines how patient perspectives influence the concepts measured and the items constructed, and aims to bridge gaps between them. Moreover, it gathers patient input to increase the suitability of the items so that they reflect patient experiences closely, facilitating the correct understanding and interpretation of patients’ responses to the items [16]. QIR provides the necessary attention to patient participation to make it a sound choice as a method for developing a PROM-PGHD.

QIR was specifically designed to optimize a set of PROM items in preparation for field testing. It was meant to develop an initial set of PROM items qualitatively and revise them by eliciting patient participation. Quantitative field testing, for example, using psychometric criteria, may then follow QIR, according to good practice guidelines [16].

Comparing QIR with the PROM development process described in the literature reveals that it closely aligns with early stage qualitative activities, that is, Stage I of the process suggested by the SAC [22], and thus with phases/steps 1 and 2 of the review paper [24] and FDA guide [10]. The QIR steps are summarized in Table 3.

**Table 3:** Activities of the qualitative item review.

Number	Step name	Activities
1	Literature review to identify existing items	Scan literature around established PROMs^a^ within target domain/s; it will guide building proposed outcome measure items. Items identified represent the range of domain-relevant experiences.
2	Binning and winnowing	Binning involves categorizing selected items according to meaning and intrinsic structure. Winnowing excludes items that do not fit target domains and characteristics of PROM being developed.
3	Item revision process	Retained items are appropriately revised to ensure they are independent, have similar contexts, concise and simple, and worded to encourage the use of available response options to reduce cognitive burden on respondents.
4	Focus groups and cognitive interviews with target patient cohort	It ensures patient input is elicited in the development of PROM item sets. It enables PROM designers to understand vocabulary and thinking processes of target group and gathers feedback on individual items. It is aimed to bridge relevant gaps between current items and target domain or concepts to be measured. It highlights other measurement areas expressed by patients that are not covered in initial item set.
5	Final item revisions	Items are revised again based on patient input gathered from previous step. Items are tested with the Lexile Analyzer (MetaMetrics, Inc) to assess readability. After revisions are completed, field testing on items may begin, to understand their quantitative characteristics.

^a^PROM: patient-reported outcome measure.

### Augmenting the Qualitative Item Review Process for the Socio-Technical Context of Person-Generated Health Data

The development of a PROM-PGHD requires socio-technical consideration of PGHD and the health technologies from which they are produced. Health-related activities of patients are influenced by the social and health context of the patient and their family and community [28]. This contributes to the complexity of what is known as the socio-technical system in health care, referring to the social system that influences and is influenced by implementations of technical systems. Thus, a socio-technical approach to health informatics interventions is crucial [29]. The health tools and technologies that patients use are most effective if they align with the patients’ goals for completing health-related activities within the context of their health conditions. Moreover, health information technology interventions need to be responsive to the biomedical realities and personal characteristics of the target patient population [28].

Therefore, in developing a PROM-PGHD, it is important to recognize two domains influencing the outcome to be measured [30,31]: the health condition and the technology category. The evaluation of PGHD’s role in self-management and clinical care should draw upon the body of knowledge from both domains [32]. There are different possible effects on health conditions in patients who use data from a web portal, a smart phone app, or a wearable sensor, just as there are different possible health effects of using data from a smartphone app in patients with diabetes, a mental illness, or asthma [1]. This is an important consideration, as the value of a PROM is dependent on its appropriateness based on the needs of the patient population [33].

Our development also factored in a key difference between the objectives of the PROMIS initiative for which the QIR was designed and the objective of PROM-PGHD. The PROMIS initiative’s item banks, that is, PROM item sets, were developed to capture patient-reported outcomes from mainstream interventions, in particular health conditions, for example, chronic diseases [16]. Meanwhile, PROM-PGHD items are meant to capture patient-reported outcomes of accessing and utilizing PGHD they themselves have produced with various types of health technology in relation to a particular health condition.

An important consideration of this socio-technical approach is that when it comes to the technology category, outcome measures may extend beyond traditional PROMs of the health condition to include self-reported measures that capture the effects of a patient’s interaction with their data, as this interactivity is designed into a type of technology. Thus, we augmented the QIR process of developing PROMs to consider both the health condition and the technology category for which a PROM-PGHD is being developed.

## Results

### A Worked Example of the Patient-Reported Outcome Measure of Utilizing Person-Generated Health Data Development Method

To illustrate how the PROM-PGHD development method may be used to develop a PROM-PGHD, a worked example is presented based on the steps presented in Table 3. This is further augmented as described above. This example demonstrates how augmenting the first QIR step guides the identification and development of items within the domains of interest and influences the development process. Each of the five steps is outlined, with references to work on each step that we have reported elsewhere. These references to papers published to date are summarized below:

- Step 1, literature review: Dimaguila et al [8].
- Analysis of Step 1 and implementation of Steps 2 and 3: Dimaguila et al [34].
- Step 4, eliciting patient input: Dimaguila et al [7].

**Figure 1:**
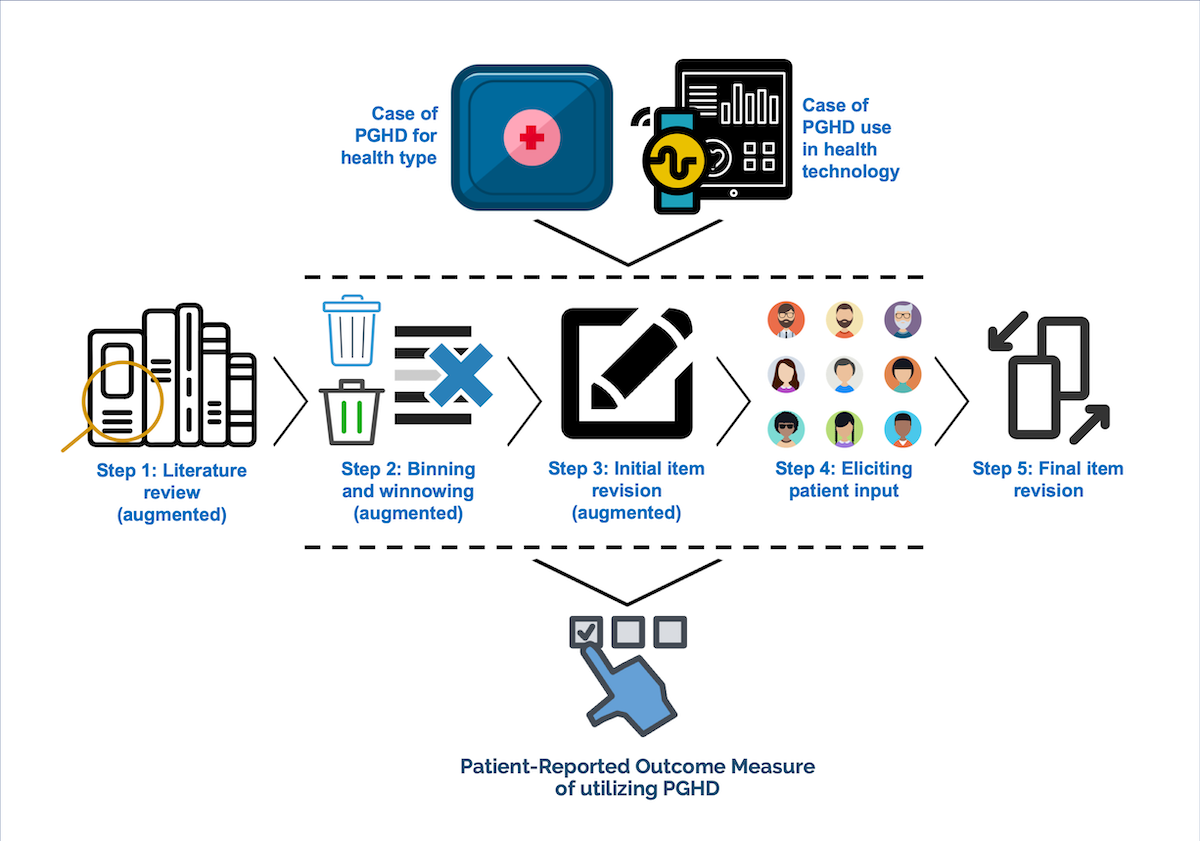
The steps of the patient-reported outcome measure of utilizing person-generated health data (PGHD) development method, which was augmented from the qualitative item review. Icon sources: Iconfinder and Flaticon.

Figure 1 outlines the steps of the PROM-PGHD development method and indicates how the socio-technical context influences the process from the beginning.

### Case Study

An exemplar PGHD use case is home-based stroke rehabilitation (the health condition) using body-tracking simulated technologies (the technology category) [8]. Stroke is a leading global cause of death and disability [35,36]. Clinical rehabilitation is lengthy and costly; thus, home-based rehabilitation may improve outcomes, and patients may prefer home-based options rather than traveling to clinics [37]. Simulated stroke rehabilitation systems, in particular using the industry-leading Kinect (Microsoft), simulate rehabilitation activities in a clinical environment in real time [38]. These systems use a video gaming console, which may be well suited for home-based rehabilitation. Patients may generate data through different forms of interaction [39-41]. More information on Kinect, for example, how it was designed and types of rehabilitation tasks available, is provided in previously published literature [42-44]. Utilizing PGHD in conjunction with such systems has the potential to generate important new evidence about the efficacy of stroke telerehabilitation. Therefore, a PROM-PGHD of Kinect-based stroke rehabilitation systems is our example of step-by-step item development.

#### Step 1: Literature Review (Augmented)

The first step, that is, literature review, is key in identifying concepts and items within the domain of interest for the PROM being developed. It identifies items representing the range of domain-relevant experiences [16].

Augmenting it to include the health condition and the technology category recognizes the socio-technical context of PGHD-enabled technologies and ensures that relevant items from both domains are included. This was implemented for the worked example, and as such, influenced the identification of outcome measures from the literature. An extensive literature review was conducted for this example combination of a health condition and a technology type detailed in Dimaguila et al [8]. The review examined the extent of PGHD utilization in studies of Kinect-based simulated rehabilitation systems for stroke and identified outcome measures from which candidate items were drawn for assessment. Outcome measures identified from papers selected in the review include the Game Experience Questionnaire [45] and the Stroke Impact Scale [46].

#### Step 2: Binning and Winnowing (Augmented)

The second step is *Binning and Winnowing*. The overall objective of the binning (ie, including) activity is to build sets of items that represent an aspect of a particular health condition, for example, walking within a physical function condition [16]. For PROM-PGHD, we endeavored to develop sets of items that instead represented reported effects of PGHD utilization [34]. This is to match the objective of PROM-PGHD. Moreover, an additional exclusion criterion was introduced for winnowing activity. Originally, this step excluded (*winnowed*) items that were too narrow, disease specific, redundant, or confusing [16]. For the purposes of the PROM-PGHD, an additional criterion was added to winnow items whose content would not be able to measure the effects of utilizing PGHD, as described here [34]. These effects include influencing interest in their care processes [2-4], and changing feelings about health status [3], and were derived from key themes that occurred in a key journal special issue on PGHD [1].

The outcome measure items identified in the previous step with consideration of the socio-technical context of the case study were assessed for appropriateness to PROM-PGHD, that is, their relevance to the reported effects of PGHD [34]. Items were winnowed according to the criteria described earlier. Retained items were binned by aligning existing items selected from Step 1, with reported effects on patients who used PGHD in controlled settings [34].

#### Step 3: Initial Item Revision (Augmented)

In the third step, that is, item revision, PROM items are revised to ensure consistency of their response options, similarity in wording contexts, conciseness and simplicity of wording, their independence from other questions, and that they encourage the use of available response options [16]. In addition, for PROM-PGHD, it may be necessary to revise some terminology used in the items, so they would better match the target health condition and technology category. Items may be worded quite generally, and revision would make them more specific to the target domains [34]. In the worked example, after Step 2, the preliminary item bank was revised to better match the target domains of Kinect-based stroke rehabilitation systems. Revisions were also conducted to address inconsistent response options and experience-recall time frames for the purpose of maintaining consistency [34]. Suggested uniform response options for the PROMIS rating scales [16] were followed.

Implementing the first step typically results in a number of diverse PROM items (eg, the question) and corresponding response options (eg, range of likelihood from agree to disagree, or a scale of 1-5) [16]. The optimal response options may vary based on the individual items they correspond with, and there is no empirical evidence suggesting that some sets of response options are clearly superior to others, that is, are consistently more accurate at capturing respondent experiences. Thus, it may be necessary to determine the response options through a consensus process with domain experts [16] or with the target patient cohort [34]. Additional response options were added to gather feedback from patients themselves in Step 4, on the appropriateness of the item response types [34]. The revised items were then grouped according to their alignment with a PGHD effect, and according to their response option types, that is, true/false statements, rating scales, and multiple-choice questions [34]. The subsequent step, which elicits patient participation, is expected to improve the suitability of the items [16].

This step resulted in a preliminary PROM-PGHD item bank, which was then presented to patients in the next step [34]. Augmenting the first step of QIR, to consider the socio-technical context of health technologies from which PGHD are produced, ensured that the outcome measures and items considered were drawn from the domains of interest, that is, the health condition and the technology category. Thus, the items that were considered for binning and winnowing, underwent initial item revision and eventually were presented to the patients for comment, covered relevant concepts from both domains [34].

#### Step 4: Eliciting the Patient Input

In this step, stroke survivors participated in focus groups and semistructured interviews, where they were asked to comment on the concepts and items of the preliminary PROM-PGHD item bank, for example, on the items’ clarity and suitability to their experience. Detailed analysis and reporting of the data collected in these studies are presented elsewhere [7]. They were also asked open-ended questions about their experience of accessing and utilizing PGHD for the purpose of gathering concepts that may not have been covered by the current items. Based on the exemplar health and technology case being investigated, the target patient cohort was stroke patients with varying levels of experience with Jintronix (Montreal, Canada), a simulated rehabilitation software system using Kinect version 2 [43] and which is FDA approved [47]. Patient recruitment was conducted at three different sites, with ethics approval granted by the Human Research Ethics Committees of Deakin University (2017-087), Austin Health (HREC/17/Austin/492), and the University of Melbourne (1852259.1).

Some of the PGHD effects previously reported in the literature were reaffirmed by the patients, for example, that PGHD access can increase engagement with the recovery process. However, patient input showed that some effects were dependent on the status of their PGHD, for example, they felt satisfaction only when their PGHD showed an improvement trend [7]. This highlights the importance of eliciting patient input to gather a richer understanding of patient-reported outcomes [12,16].

#### Step 5: Final Item Revision

This step includes improving the PROM-PGHD items’ accuracy in representing the perspectives and experiences of the target patient cohort, and their suitability and clarity. In the worked example, revisions took the form of direct changes to the wording of the items, reduction or addition of response options or scales, and reduction or addition of outcome items. For example, we have learned from our discussions with patients that our preliminary PROM-PGHD lacks an item to measure *levels of frustration*, which patients experience when they see their PGHD fluctuate, that is, indicators of their health status that go up and down over time [7]. The current PROM-PGHD was therefore revised to add *levels of frustration* as an outcome measure.

Finally, the items were run through the MetaMetrics Lexile analyzer (MetaMetrics, Inc) to assess their readability based on sentence length and the commonness of words. This provides an extra layer of assessment to determine if any items could be problematic during implementation, and to conduct revisions as necessary to improve readability [16]. The full revision related to this step in our worked example, to be reported elsewhere, will prepare the PROM-PGHD item set for quantitative field testing [16].

## Discussion

### Relevance

This paper has argued that a PROM of utilizing PGHD is necessary to provide clearer evidence about the value of implementing related health technologies. PROMs-PGHD would provide a systematic way for patients to gain insights into the health effects they experience from utilizing their PGHD. PROMs-PGHD could also be included routinely as part of the patient record, where PGHD are produced within a patient’s care plan. This is similar to how PROMs in general are used together as a set of performance measures to assess the performance of health entities and the services they provide [48,49]. As such, PROMs-PGHD could inform strategies for improving health outcomes.

As highlighted, PROMs-PGHD would fill an evidence gap and promote participatory health by recognizing the value of the patient experience when considering the use and effect of PGHD and the technologies they are produced from. They might generate more evidence about the clinical effectiveness and cost-effectiveness of PGHD-enabled technologies to aid clinicians in choosing appropriate health technologies, and for patients to understand how certain health technologies affect their health management. Moreover, PROMs-PGHD could guide technology designers in developing PGHD-enabled technologies that are more inclusive of patient perspectives, similar to how PROMs could improve the design of clinical registries [15]. Ultimately, PROMs-PGHD could contribute to building evidence-based practice in clinical work with PGHD and facilitate the creation of relevant clinical guidelines.

This paper described, and illustrated via a worked example, a method for developing a PROM-PGHD. The method was guided by an established PROM development process and a participatory health paradigm. As a result, it followed a step-wise approach of involving patients, which iteratively influences the resulting items of the PROM-PGHD as it is developed. Participatory approaches such as this can generate a rich, deep understanding of the effects of a health technology intervention [12] and ensures that the patient perspective is embedded into the resulting PROM-PGHD, which is central to the value of PROMs [14].

The PROM-PGHD development method follows the best practice as it is distilled from the literature, adding to its credibility in producing legitimate measures of patient-reported outcomes. In addition, its consideration of the socio-technical context of health technology interventions increases its sensitivity to personal characteristics and the physiological and health-related factors affecting the target patient cohort [28]. The recognition of the two domains inherent in health informatics [30,31], that is, health condition and technology category, increases the appropriateness of the resulting PROM-PGHD for assessing the effects experienced by the patient cohort.

This worked example has shown that the PROM-PGHD development method is meaningfully applied to a PGHD-enabled technology category used in a specific health condition. It has identified existing PROM items relevant to the chosen domains: stroke and Kinect-based simulated rehabilitation technology. This helps ensure that the resulting PROM-PGHD is reflective of the experiences of patients who are using a technology within the context of their health condition. This allows the PROM-PGHD development method to be used in other cases where health technologies are implemented in health conditions.

It is important for practitioners and developers of health technologies to prioritize the patient’s perspective and to be sensitive to how PGHD may affect people differently [8,9]. Future studies should therefore apply the PROM-PGHD development method in other relevant contexts where it may be important to understand how the health condition and technology category have interrelated effects on patients’ outcomes from using PGHD [31,32]. Revising and retesting the resulting item banks in clinical samples would also increase the validity of the method [50], and it could be valuable to further explore how other socio-technical factors, such as health literacy, influence responses to the PROM-PGHD.

### Limitations

One limitation of the QIR process [16], and thus with the PROM-PGHD development method, is the necessity to change the existing items selected from the literature review. The changes considered to be minor are conducted during the item revision steps. They are necessary to improve the uniformity of the response options that are designed to be read and interpreted by patients [16,34]. However, this process is not believed to substantially alter any existing outcome measure items. Moreover, the subsequent steps that elicit patient participation are expected to improve the suitability of the items [16].

### Conclusions

This paper highlights the need for a systematic way of measuring the effects of PGHD on the health of people who utilize them. A method was presented for developing such a measure, called PROM-PGHD, based on best practice within the participatory health paradigm and in consideration of the socio-technical context of PGHD utilization. A new PROM-PGHD development method was illustrated through the example of stroke survivors using Kinect-based poststroke simulated rehabilitation technologies. It was shown that the method can be applied successfully to develop an initial set of items from the domains of the health condition and technology category. This method may be applied to other cases that combine a health condition and a technology category, and thus, this method could have broader relevance for EBP in clinical work with PGHD. Future studies should apply the PROM-PGHD development method within other relevant socio-technical contexts, and revise and retest the resulting item banks.

## Abbreviations

EBP: evidence-based practice
FDA: United States Food and Drug Administration
PGHD: person-generated health data
PROM: patient-reported outcome measure
PROM-PGHD: patient-reported outcome measure of utilizing person-generated health data
PROMIS: Patient-Reported Outcomes Measurement Information System
QIR: qualitative item review
SAC: Scientific Advisory Committee of Medical Outcomes Trust
